# Q fever endocarditis masquerading as Mixed cryoglobulinemia type II. A case report and review of the literature

**DOI:** 10.1186/1471-2334-6-32

**Published:** 2006-02-23

**Authors:** Petros I Rafailidis, Spiros P Dourakis, Christos A Fourlas

**Affiliations:** 1 Second Academic Department of Internal Medicine of the University of Athens, Hippokration General Hospital, Athens, Greece

## Abstract

**Background:**

The clinical manifestations of Q fever endocarditis are protean in nature. Mixed cryoglobulinemia type II is rarely a facet of the presenting clinical manifestations of Q fever endocarditis.

**Case presentation:**

We report a case of a 65-year-old pensioner with such an association and review the literature. As transesophageal echocardiograms are usually normal and blood cultures are usually negative in Q fever endocarditis, many of the manifestations (fever, rash, glomerulonephritis/evidence of renal disease, low serum C4 complement component, presence of mixed type II cryoglobulin, constitutional symptoms as arthralgias and fatigue) can be attributed to Mixed cryoglobulinemia type II per se. The use of Classic Duke Endocarditis Service criteria does not always suffice for the diagnosis of Q fever.

**Conclusion:**

The application of the modified criteria proposed by Fournier et al for the improvement of the diagnosis of Q fever endocarditis will help to reach the diagnosis earlier and thus reduce the high mortality of the disease. We would like to stress the importance of ruling out the diagnosis of Q fever endocarditis in cases of mixed type II cryoglobulinemia.

## Background

Clinical manifestations of Q fever endocarditis are protean in nature. Mixed cryoglobulinemia type II is rarely a facet of the presenting clinical manifestations of Q fever endocarditis.

## Case presentation

A 65-year-old pensioner was admitted to our hospital in May 2000 because of a two-month history of fever up to 38°C. This was accompanied by fatigue, arthralgia, muscle weakness and night sweating. Seven months ago he reported a similar clinical constellation, which lasted approximately three weeks, and at that time a presumptive diagnosis of infection due to *Brucella melitensis *was made elsewhere, based on the findings of ELISA (increased IgM antibody titre and a fourfold increase of IgG antibody titre in samples taken at two different periods). He received ciprofloxacin for presumed brucellosis for a total of ten weeks and remained asymptomatic for four months. During the previous 8 months the patient had noticed evanescent skin eruptions of red to purple papules, which remained only for a few days each time but left areas of brown hyperpigmentation.

Past medical history: Fifteen years ago he had surgery for a stenosed mitral valve and a prosthetic Starr-Edwards was inserted. He is on asenocoumarol and digoxin since. He was neither smoking nor consuming alcohol.

On examination: Blood pressure: 120/70 mm Hg. Temperature: 38°C. Pulse: 70 per minute irregularly irregular. Respirations: 12 per minute. He looked emaciated and ill though not in acute stress. His sclerae were pale. A scar of the previous cardiothoracic operation was evident. The first heart sound had a metallic resonance. A soft systolic 1–2/6 murmur could be heard at the apex and the lower left sternal edge. The liver was palpable 2 cm below the right hypochondrium. The tip of the spleen was palpable during inspiration. A palpable purpuric rash was present on the legs, only for roughly two days, as were areas of hyperpigmentation (figure).

Investigations revealed a normochromic normocytic anemia (hemoglobin: 11,4 gr/L) accompanied by decreased renal function (serum creatinine: 159,1 μmol/L), an ESR of 125 mm/ 1^st ^hour and hyperglobulinemia (5,8 gr/dl).

Serum protein electrophoresis revealed increased γ globulin: 41,4% (normal: 11,0–22,2%), decreased albumin: 34% (normal: 51,2–63%) and normal α_1 _globulin, α_2 _globulin and β globulin. Quantitative estimation showed: IgG: 4930 mg% (normal: 694–1618 mg%), IgA: 821 mg% (normal: 69–378 mg%), IgM: 533 mg% (normal: 60–263 mg%), κ: 3270 mg% (normal: 574–1276 mg%) and λ: 1740 mg% (269–638 mg%). Rheumatoid factor was markedly increased at 1430 IU/L (normal: <20 IU/L). Cryoglobulins were present in the serum and the corresponding cryocrite was 3,3%. In particular immunofixation of serum proteins at 37°C revealed: a. an IgM κ paraprotein in the gamma globulin region and b. a diffuse polyclonal increase of IgG, which corresponded to diffuse κ chains. Serum complement showed a borderline low normal C_4_: 20,5 mg/dl (normal: 20,5–49 mg/dl) whilst C_3 _was normal. Urine dipstick and microscopy disclosed: protein +, hemoglobin +++, 2–3 leucocytes per optical field, 30–40 RBC per optical field, rare hemorrhagic casts, rare RBC casts and mixed cellular casts.

As the clinical manifestations and laboratory data of our patient dictated, we pursued ruling out infective endocarditis and various other infectious causes of mixed type II cryoglobulinemia. Blood and urine cultures were repeatedly normal. Two transesophageal echocardiographs during his hospitalization performed by an experienced board certified cardiologist did neither show vegetations nor any valve malfunction. Wright agglutination test, VDRL and serological tests for HBV, HCV, EBV, CMV and HIV were all negative. HCV RNA was negative in the serum, the cryoprecipitate and the supernatant. Blood cultures and bone marrow cultures were negative for Brucellae, Mycobacteria and Leishmaniae. Blood was tested for Brucella DNA by PCR and was negative. Abdominal ultrasound revealed reduced kidney size (96 mm) and hepatosplenomegaly. Computed tomography of the thorax and abdomen was otherwise normal. A whole body scintigraphy with gallium 67 was normal.

Based on the findings of the purpuric rash of the lower limbs, the borderline low serum C_4 _complement component, the findings of renal disease (increased creatinine, hematuria, red blood cells casts), the presence of mixed type II cryoglobulinemia (as already mentioned, immunofixation of serum proteins at 37°C revealed an IgM κ paraprotein in the gamma globulin region and a diffuse polyclonal increase of IgG, which corresponded to diffuse κ chains) and the normal transesophageal echocardiograms and negative tests for viruses (HCV, HBV, EBV, CMV) a working diagnosis of idiopathic mixed type II cryoglobulinemia was made. Strict need for anticoagulation (due to his prosthetic mitral valve) precluded biopsy of afflicted organs. The patient was prescribed a 15 mg prednisolone dose per os daily.

He experienced temporary relief of his symptoms for a period of three weeks. He subsequently presented with dyspnoea, relapse of fever and a worsened holosystolic 4/6 murmur at the cardiac apex, which radiated to the axilla. Transesophageal ultrasound at this time showed paravalvular leak of the prosthetic mitral valve. Antibodies against *Coxiella burnetii *[using indirect microimmunofluorescence with the Q fever reagent of MRL (now Focus)] were positive phase I IgG: 1/64000, phase I IgM: 1/1024. Phase II IgM antibodies were negative and phase II IgG positive at 1/64000. Polymerase chain reaction for the detection of *Coxiella burnetii *DNA in blood was positive. A Trans-PCR was performed using primers Trans1 (5'-TAT GTA TCC ACC GTA GCC AGT C-3'), Trans2 (5'-CCC AAC AAC ACC TTC TTA TTC-3') which flanked a 687-bp transposonlike repetitive region of the *C. burnetii *genome. Two internal primers were used as well namely Trans3 (5'-GTA ACG ATG CGC AGG CGA T-3') and Trans4 (5'-CCA CCG CTT CGC TCG CTA-3'). Serology was negative for *Chlamydia psittaci, Chlamydia pneumoniae, Chlamydia trachomatis, Legionella pneumophila, Rickettsia mooseri *and *Rickettsia conorii*. After two weeks of treatment with doxycycline 100 mg bd per os and hydroxychloroquine 200 mg bd per os he had his Starr-Edwards valve replaced by a prosthetic ATS 25 mmHg mitral valve. Today approximately five years after the operation he remains in excellent health with no cryoglobulins detected and with a dramatic decrease in antibody titres against *Coxiella burnetii *while on the same regimen, which was recently discontinued. The duration of treatment with doxycyclin-hydroxychloroquine was 5 years.

## Discussion

Clinical manifestations of chronic Q fever are endocarditis, isolated fever, osteoartricular infections, chronic hepatitis and rarely chronic pericarditis, skin lesions, interstitial lung fibrosis and pseudotumor of the lung [1,2]. These protean ailments have led to the classification of chronic Q fever into 1. Q fever endocarditis 2. Chronic Q fever with underlying cardiac or vascular disease but with no evidence of endocarditis 3. Chronic Q fever without cardiovascular involvement [3].

The incidence of endocarditis due to *Coxiella burnetii *in the general population is approximately 1 per 10^6^. Early diagnosis is hampered by the variation of its clinical manifestations. It is a severe and often fatal disease [4]. Mortality approaches 100% without treatment, whilst therapy reduces this percentage to 10–24% [1]. Endocarditis is consisting 16–73% of all chronic Q fever, being so the major clinical representative. A lag of 12–24 months from onset to diagnosis of Q fever endocarditis (8 months in our patient's case) is not unusual due to the non-specific symptoms and signs and because vegetations are either to small to be depicted on echocardiography or are completely absent. In a series of 168 patients with *C. burnetii *endocarditis only 40% had vegetations [5]. This was also initially the case in our patient where during his hospitalisation two transesophageal echocardiograms performed by an expert cardiologist did not show any abnormality.

The other major criterion (namely two separate blood cultures according to classic Duke Endocarditis Service criteria) for the diagnosis of infectious endocarditis could not be met in our patient, as culture of *Coxiella burnetii *cannot be achieved in most routine laboratories of the world because of the biohazard associated with its culture. As transesophageal echocardiograms are usually normal and blood cultures are usually negative in Q fever endocarditis, a modification of the diagnostic criteria by the Duke Endocarditis Service has been proposed: Q fever serology (1/800 antiphase I immunoglobulin G cut off) and a single blood culture of *Coxiella burnetii *should be regarded as major criteria and not as minor ones [4]. This amendment helped to improve the diagnosis of Q fever endocarditis in as much as 20% of the cases in the described case series [4].

Cryoglobulins are detected in 84–95% of all cases of infectious endocarditis and in less than 5% in Q fever endocarditis [6]. Q fever endocarditis can mimic the clinical syndrome of mixed cryoglobulinemia type II. This happens because many of the characteristics are shared by the two diseases (fever, rash, glomerulonephritis/evidence of renal disease, low C4 complement component, presence of mixed type II cryoglobulin, constitutional symptoms such as fatigue, arthralgia). As transesophageal echocardiograms are usually normal and blood cultures are usually negative in Q fever endocarditis, many of these clinical manifestations can be attributed to Mixed cryoglobulinemia type II per se [[7-11]]. Thus problems of differential diagnosis arise.

We reviewed the five other cases of the literature where such issues of differential diagnosis between mixed cryoglobulinemia type II and Q fever endocarditis emerged and caused a delay in diagnosis [see Additional file 1]. Our patient and the other five patients with mixed cryoglobulinemia in association with Q fever endocarditis described in the literature, have the following features in common: presence of a prosthetic valve, normal echocardiograms at least at an initial stage of the disease, and presence of a rash (mainly purpuric) on the lower limbs. Two of these patients were unnecessarily treated with some form of immunosuppressive therapy (steroids, cyclophosphamide, splenectomy), as was the case for our patient as well. The common denominator is that eventually diagnosis was reached by the use of serology for *Coxiella burnetii*, which was positive in all reported cases. A repeat echocardiogram showed abnormalities in two of the five cases, while no data are given for two cases. We want to stress that in our patient even a second transesesophageal echocardiogram was negative. We think that by having applied the modified Duke Endocarditis Service criteria in all these cases (including ours), which include serology against *Coxiella burnetii *as a major and not as a minor criterion, diagnosis would have been reached earlier.

In the course of his disease, our patient underwent an extensive workup in order to rule out the commonest causes of mixed type II cryoglobulinemia. Among them *Hepatitis C virus *infection having a dominant role [12] was excluded not only by serology but also with a search for HCV RNA in the cryoprecipitate and its supernatant. Because of his prior presumed brucellosis we searched meticulously for this possibility. We think that this previous diagnosis, which was based on positive ELISA assay, was a serological cross-reaction with *Coxiella burnetii*. Whilst *Coxiella burnetii *has been reported in the past to cross-react with a variety of organisms (such as *Rickettsia ricketsii, Ehrlichia chaffeensis, Bartonella quintana, Bartonella henselae, L. pneumophila, L. micdadei, Chlamydia spp *and *Human granulocytic ehrlichiosis agent*) [13], this is to the best of our knowledge the first case in the literature where such an association exists in conjunction to *Brucella melitensis*. We believe that his prior response to ciprofloxacin can be explained by the fact that this antibiotic has good efficiency not only for *Brucella spp*. infections (though relapses are not uncommon) but also for *Coxiella burnetii *infections. *Bartonella quintana and Bartonella henselae *are important causes of culture negative endocarditis with vegetations found in 84% of these patients [5].

Therapy of chronic Q fever prosthetic valve endocarditis involves surgical replacement of the infected valve, as it is not likely for the patient to respond to conservative means alone [14]. The cardiothoracic operation per se can lead to recurrence of the disease [15,16]. The two prevailing regimens for treatment involve doxycycline (200 mg) with hydroxychloroquine (200–800 mg) for eighteen months or doxycycline at the same dose plus ofloxacin (600 mg) for four years [17]. Many believe that treatment is needed for a lifetime, since late recurrences occur in as many as 50% after treatment discontinuation [3,17]. Our patient was on the first regimen (dose for hydroxychloroquine 400 mg) until recently and is doing extremely well. In addition no cryoglobulins are detected, the IgG titer against phase I has fallen to 1/2048 and the IgG titer against phase II has fallen to 1/2048.

## Conclusion

In cases of mixed type II cryoglobulinemia, chronic infection due to *Coxiella burnetii *should be ruled out, while modified Duke Endocarditis Service criteria will help to pick up the diagnosis earlier in difficult cases.

## Competing interests

The author(s) declare that they have no competing interests.

## Authors' contributions

PIR, SPD, CAF drafted the manuscript. All authors read and approved the final manuscript.

**Figure 1:**
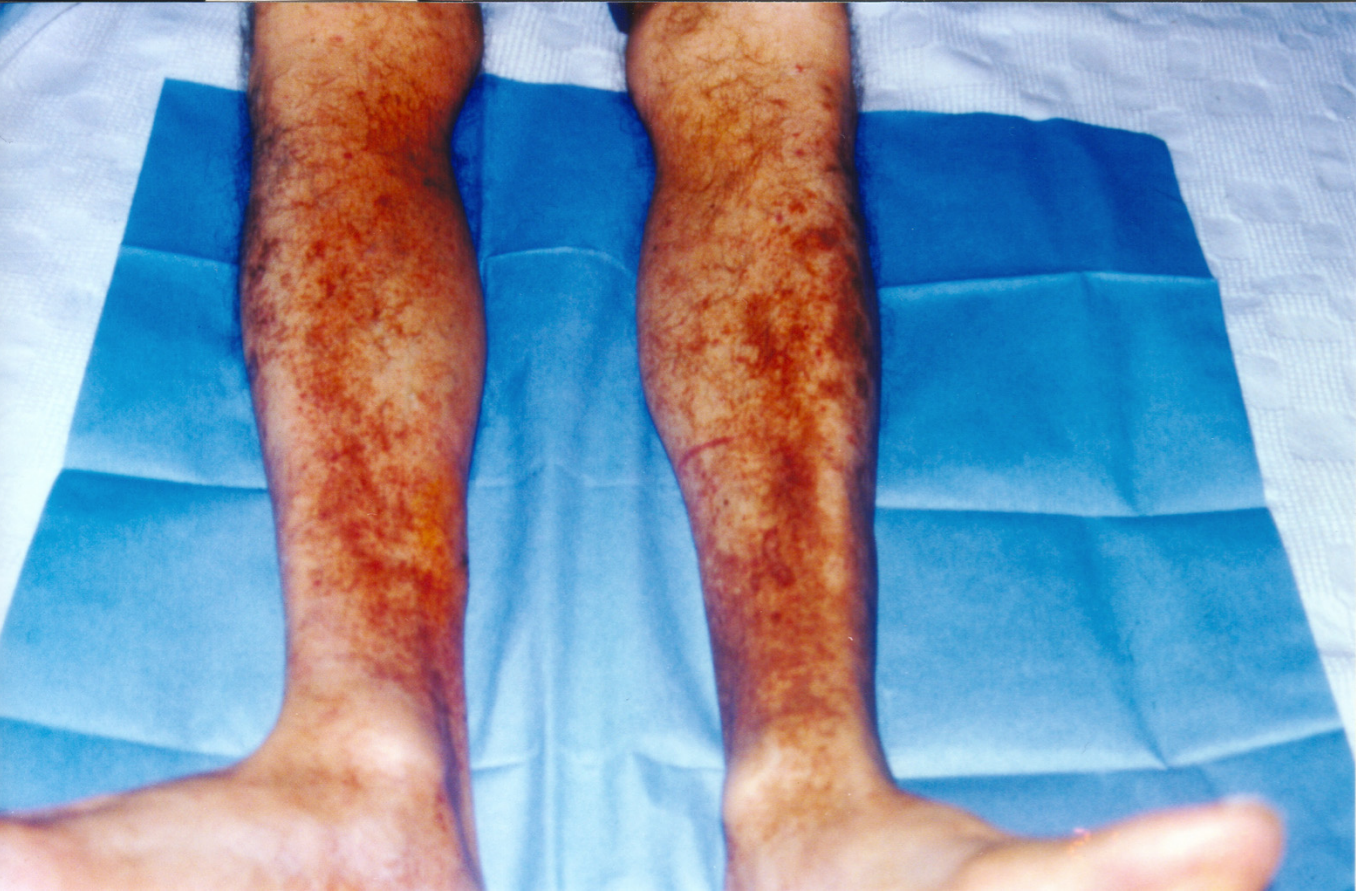
Purpuric rash on the patient's legs with areas of hyperpigmentation.

## Pre-publication history

The pre-publication history for this paper can be accessed here:



## Supplementary Material

Additional File 1 Cases of mixed cryoglobulinemia in association with Q fever endocarditis presented in the literature. Click here for file

## References

[B1] Raoult D, Dupont-Tissot H, Foucault C, Gouvernet J, Fournier PE, Bernit E, Stein A, Nesri M, Harle JR, Weiller PJ (2000). Q Fever 1985–1998 Clinical and Epidemiologic Features of 1383 Infections. Medicine.

[B2] Siegman-Igra Y, Kaufman O, Keysary A, Rzotkiewicz S, Shalit I (1997). Q Fever Endocarditis in Israel and a Worldwide Review. Scand J Infect Dis.

[B3] Stein A, Raoult D (1995). Q fever endocarditis. Eur Heart J.

[B4] Fournier PE, Casalta JP, Habib G, Messana T, Raoult D (1996). Modification of the Diagnostic Criteria Proposed by the Duke Endocarditis Service to Permit Improved Diagnosis of Q Fever Endocarditis. The American Journal of Medicine.

[B5] Houpikian P, Raoult D (2005). Blood culture-negative endocarditis in a reference center. Etiologic diagnosis of 348 cases. Medicine.

[B6] Brouqui P, Dupont-Tissot H, Drancourt M, Berland Y, Etienne J, Leport C, Goldstein F, Massip P, Micoud M, Bertrand A, Raoult D (1993). Chronic Q fever. Arch Intern Med.

[B7] Torley H, Capell H, Timbury M, McCartney C (1989). Chronic Q fever with mixed cryoglobulinaemia. Ann Rheum Dis.

[B8] Enzenauer RJ, Arend WP, Woodruff Emlen J (1991). Mixed cryoglobulinemia associated with chronic Q- fever. J Rheumatol.

[B9] Coponat-Vacher H, Dussol B, Raoult D, Casanova P, Berland Y (1996). Proliferative glomerulonephritis revealing chronic Q fever. Am J Nephrol.

[B10] Ghassemi M, Agger WA, Vanscoy RE, Howe GB (1999). Chronic sternal wound infection and endocarditis with Coxiella burnetii. Clin Infect Dis.

[B11] Granel B, Genty I, Serratrice J, Rey J, Disdier P, Raoult D, Weiller PJ (1999). Livedo reticularis revealing a latent infective endocarditis due to Coxiella burnetii. J Am Acad Dermatol.

[B12] Bichard P, Qunanian A, Girard M, Baccard C, Rolachon A, Renversez JC, Cordonier D, Seigneurin JM, Debru Jl, Zarski JP (1994). High prevalence of Hepatitis C virus RNA in the supernatant and the cryoprecipitate of patients with essential and secondary type II mixed cryoglobulinemia. Journal of Hepatology.

[B13] Graham JV, Baden L, Tsiodras S, Karchmer AW (2000). Q fever endocarditis associated with extensive serological cross-reactivity. Clin Infect Diseases.

[B14] Berbari EF, Cockerill FR, Steckelberg JM (1997). Infective Endocarditis due to Unusual or Fastidious Microorganisms. Mayo Clin Proc.

[B15] Lev BI, Shachar A, Segev S, Weiss P, Rubinstein E (1988). Quiescent Q fever endocarditis exacerbated by cardiac surgery and corticosteroid therapy. Arch Intern Med.

[B16] Marrie TJ, Cunning J, Durnford P (1992). Reactivation of Q Fever following cardiac surgery. Eur Clin Microbiol Infect Dis.

[B17] Raoult D, Houpikian P, Tissot Dupont H, Riss JM, Arditi-Djiane J, Brouqui P (1999). Treatment of Q fever endocarditis: comparison of 2 regimens containing doxycycline and ofloxacin or hydroxychloroquine. Arch Intern Med.

