# Supplementary material for: Q fever endocarditis masquerading as Mixed cryoglobulinemia type II. A case report and review of the literature
Source: BMC Infect Dis. 2006 Feb 23;6:32. doi: 10.1186/1471-2334-6-32 (PMC1397847; doi:10.1186/1471-2334-6-32)
Supplement: Additional File 1 — Cases of mixed cryoglobulinemia in association with Q fever endocarditis presented in the literature. [file 1471-2334-6-32-S1.doc]

Reference Age Prosthesis Echo Repeat Echo Delay in diagnosis Rash of lower limbs Steroids/Other Therapy Serology PCR Valve Replacement

after diagnosis

6 66 Aortic valve - (TEE) + 36 months Yes +/splenectomy + + No

7 47 Mitral valve - - 6 months Yes -/- + NP No

8 41 Mitral valve - NR 22 months Yes +/cyclophosphamide + NP Yes

9 69 Mitral valve - (TTE) + (TEE) 10 months No -/- + NP No

10 54 Mitral valve - (TEE) NR NR Yes (livedo reticularis -/- + NP No

instead of purpuric)

Additional file 1: Table. Cases of mixed cryoglobulinemia in association with Q fever endocarditis presented in the literature.

TEE: Transesophageal Echocardiography, TTE: Transthoracic echocardiography, NR: Not Reported, NP: Not Performed
